# OP60 National Horizon Scanning System In Brazil: Current Actions And Outputs

**DOI:** 10.1017/S0266462325101189

**Published:** 2025-12-29

**Authors:** Ana Carolina de Freitas Lopes, Thais Conceição Borges, Aramis Tupiná Alcântara de Moreira, Karine Medeiros Amaral, Munique Gonçalves Guimaraes, Viviane Del Lama Cardoso Salas, Barbara Pozzi Ottavio, Luciene Bonan

## Abstract

**Introduction:**

Horizon scanning (HS) initiatives in Brazil began in 2008. Since then, the implementation of a national HS system has shown great evolution and expansion, especially after the creation of the National Committee for Health Technology Incorporation (CONITEC), which absorbed it in 2011. This work aimed to present Brazil’s National Horizon Scanning System, conducted by CONITEC, and its most recent results.

**Methods:**

We developed an experience report, using mixed methods, with a descriptive analysis of HS activities conducted nationally and quantification of documents produced between December 2023 and November 2024 by CONITEC.

**Results:**

HS reports developed by CONITEC currently support the decision-making processes regarding the: (i) incorporation of technologies into the public health system (90 reports); (ii) granting of new or emerging technologies by court order (five alerts); (iii) prioritization of topics for the elaboration or update of national clinical practice guides (28 topics); (iv) national preparation in case of public health emergencies (e.g., COVID-19 and mpox) (two documents); (v) innovation and national development (five brief syntheses); and (vi) financing of research with health technologies (one document).

**Conclusions:**

The implementation of a national HS system in Brazil has evolved significantly and today it represents an important source of evidence that supports strategic decisions for Brazil’s public health system. From December 2023 to November 2024, CONITEC produced 131 HS studies to support decision-making in six different processes within the public health system.

